# *Agrimonia coreana* Extract Exerts Its Therapeutic Effect through CRAC Channel Inhibition for Atopic Dermatitis Treatment

**DOI:** 10.3390/ijms25168894

**Published:** 2024-08-15

**Authors:** Jintae Kim, Ji Min Lee, Su Jin Park, Yu Ran Nam, Seong Woo Choi, Joo Hyun Nam, Hyun Jong Kim, Woo Kyung Kim

**Affiliations:** 1 Channelopathy Research Center (CRC), Dongguk University College of Medicine, 32 Dongguk-ro, Ilsan Dong-gu, Goyang 10326, Gyeonggi-do, Republic of Koreasujin900710@gmail.com (S.J.P.); ferinus@gmail.com (J.H.N.); 2 Department of Physiology, Dongguk University College of Medicine, 123 Dongdae-ro, Gyeongju 38066, Gyeongsangbuk-do, Republic of Korea; 3 CiPA Korea Inc., Ilsan Seo-gu, Goyang 10911, Gyeonggi-do, Republic of Korea; youngshine80@naver.com; 4 Department of Internal Medicine, Graduate School of Medicine, Dongguk University, 27 Dongguk-ro, Ilsan Dong-gu, Goyang 10326, Gyeonggi-do, Republic of Korea

**Keywords:** Atopic dermatitis, *Agrimonia coreana*, CRAC channel, T lymphocyte

## Abstract

Atopic dermatitis (AD) is a common allergic inflammatory skin condition marked by severe itching, skin lichenification, and chronic inflammation. AD results from a complex immune response, primarily driven by T lymphocytes and environmental triggers, leading to a disrupted epidermal barrier function. Traditional treatments, such as topical corticosteroids, have limitations due to long-term side effects, highlighting the need for safer alternatives. Here, we aimed to show that *Agrimonia coreana* extract (AC_ext_) can be used in treating AD-related dermatologic symptoms. AC_ext_ could inhibit CRAC (Calcium Release-Activated Calcium) channel activity, reducing Orai1/CRAC currents and decreasing intracellular calcium signaling. This inhibition was further confirmed by the reduced IL-2 levels and T cell proliferation upon AC_ext_ treatment. In a mouse model of AD, AC_ext_ significantly ameliorates symptoms, improves histological parameters, and enhances skin barrier function, demonstrating its potential for treating AD.

## 1. Introduction

Atopic dermatitis (AD) is an inflammatory skin disease with a global increase in prevalence most seen in children. AD presents eczema-like symptoms including itching, which can lead to skin lichenification and chronic inflammation due to scratching [1], and these symptoms significantly impact the quality of life of patients. AD arises from a complex immune response disorder, particularly driven by various reactions of T lymphocytes, often related to environmental triggers such as IgE-mediated sensitization to food and environmental allergens. This condition can result in a chronic, relapsing form of skin inflammation resulting in a disturbance of the epidermal-barrier function [2]. Topical corticosteroids have long been considered as the mainstay of treatment for AD. However, due to the side effects associated with their long-term use, there is an increasing demand for safe and effective alternative therapies that can alleviate the symptoms in the long term without severe adverse effects.

In traditional Korean medicine, various native plants have been used for centuries to treat skin diseases. One such plant is *Agrimonia coreana* Nakai, which is a unique species in the Rosaceae family, native to Korea, and traditionally used for treating various inflammatory conditions. The *Agrimonia* genus has been utilized in herbal medicine to treat various diseases, including malaria and dysentery, and also used for hemostasis [3,4]. Extracts from *Agrimonia* species contain hundreds of chemical compounds exhibiting diverse pharmacological activities, including anti-inflammatory and anti-allergic effects [5,6,7]. Specifically, extract from *Agrimonia* leaves is also known to be effective for restoring skin barrier [8]. Among those various species, our research team has found that *Agrimonia coreana* Nakai could effectively inhibit CRAC (Calcium Release-Activated Calcium) channels, which is related to the anti-inflammatory effect [5].

The CRAC channel plays a crucial role in orchestrating the inflammatory response cascade. When external harmful stimuli such as pathogens or tissue damage are detected, antigen-presenting cells activate the T cell receptor (TCR). This activation triggers the hydrolysis of phosphatidylinositol 4,5-bisphosphate (PIP2) into diacylglycerol (DAG) and inositol 1,4,5-trisphosphate (IP3). IP3 binds to its receptor (IP3R) on the endoplasmic reticulum (ER), releasing stored calcium into the cytosol and depleting ER calcium levels. This depletion causes stromal interaction molecule 1 (STIM1) to accumulate at ER-plasma membrane junctions, activating the calcium release-activated calcium channel protein 1 (Orai1) pore unit and forming the CRAC channel, which facilitates calcium influx into the cytosol. This process, known as store-operated calcium entry (SOCE) [9,10,11] is crucial for increasing intracellular calcium levels, which in turn triggers downstream immunologic responses, including cytokine secretion, cell activation, and lymphocyte proliferation [12,13]. These mechanisms are essential for mounting an effective immune response against pathogens or resolving tissue damage [14,15].

One significant aspect of CRAC channel function is its involvement in the Orai/NFAT signaling pathway, which has been linked to skin itching in AD. Alterations in the Orai1, which regulates calcium influx, may be associated with AD [16,17]. However, the relationship of Orai or CRAC-related calcium signaling in AD pathology is not fully understood yet.

Based on this, our study focuses on the scientific validation of the efficacy of *Agrimonia coreana* Nakai as a potential treatment for AD. We seek to demonstrate that the extract of *Agrimonia coreana* Nakai inhibits excessive T cell activation in an AD model by targeting the CRAC channel, thereby reducing skin symptoms and immunologic responses, to establish *Agrimonia coreana* extract as a safe and effective treatment alternative for AD.

## 2. Results

### 2.1. Agrimonia Coreana Inhibits CRAC Channel Activity

**Figure 1:**
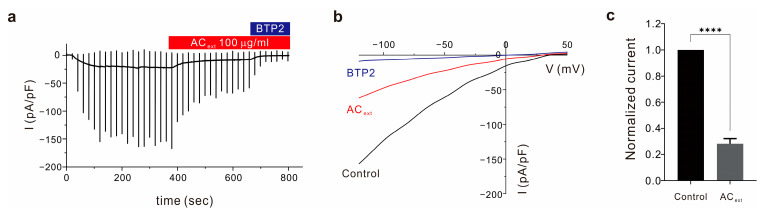
AC_ext_ inhibits the CRAC channel current in whole cell recording. (**a**) The whole-cell voltage-clamp experiments show the time course of I_CRAC_ in repeated ramp pulses between −150 mv and 50 mv. After current is stabilized, the extracellular solution is changed to *Agrimonia coreana*- (100 μg/mL) mixed extracellular solution to show inhibitory effect. BTP2 is used to show complete inhibition of the CRAC channel for normalization. (**b**) Representative current–voltage (IV) relationship curve of the I_CRAC_ with or without AC_ext_ and BTP2. (**c**) Normalized maximum current at −150 mV shows inhibitory effects of AC_ext_ (Student’s *t*-test, **** *p* < 0.0001).

In previous research, we confirmed that *Agrimonia coreana* inhibits CRAC channel activity [5]. Here, we also purified and concentrated 70% ethanolic extract from *Agrimonia coreana* with minor modification (See Section 4). To verify the *Agrimonia coreana* extract (AC_ext_) has the same effects as in the previous studies, we performed patch clamp experiments and confirmed similar inhibitory effects. When the 100 μg/mL extract was mixed into the extracellular bath solution, the recorded current was decreased (Figure 1a). After current stabilization, the current-voltage relationship curve shows lower conductivity in the AC_ext_-treated cells (Figure 1b). The normalized maximum current value treated with AC_ext_ was reduced to 28.2 ± 0.39% (mean ± SEM) of the original current (Figure 1c).

This inhibitory effect of *Agrimonia coreana* on calcium entry confirms that this extract can reduce further cascade signaling caused by increased intracellular calcium. This is related to several cellular functions, including the immunologic response in T lymphocytes.

### 2.2. T lymphocyte Proliferation Is Inhibited by Agrimonia coreana Extract Dose Dependently

To demonstrate that the immunologic response related to CRAC channels can be regulated using AC_ext_, we exposed activated T cells to AC_ext_ and observed the reaction. After the T cells had been activated using CD3 and CD28 antibodies and proliferation increases, we compared the effect of vehicle, BTP2 (CRAC channel inhibitor), and AC_ext_, along with non-activated T cells.

**Figure 2:**
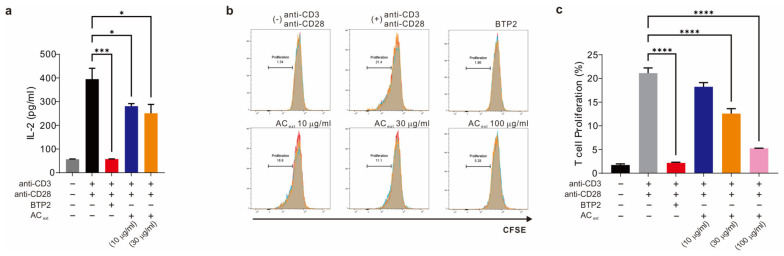
AC_ext_ inhibits activation and proliferation of T lymphocytes. (**a**) Inhibition of T cell activation function is measured by the decrease of IL-2 secretion (**b**) Cell proliferation of human CD4+ T cells were measured by flow cytometry analysis of cells stained with CFSE and cultured for 3 days after stimulation with anti-CD3 and anti-CD28. BTP2, a CRAC inhibitor at 10 μM, served as the negative control. (**c**) Multiple comparison of T cell proliferation rate (ANOVA with post-hoc test, * *p* < 0.05, *** *p* < 0.001, **** *p* < 0.0001).

For measuring the inhibitory effects, we checked Interleukin-2 (IL-2) levels in the presence and absence of AC_ext_ treatment, as IL-2 is a key indicator of T cell proliferation and activation [18]. Our results showed that with the treatment, IL-2 levels were reduced in both 10 µg/mL and 30 µg/mL concentrations (Figure 2a,b). To further examine the proliferation rate of T lymphocytes, we conducted flow cytometry analysis of T cells after carboxyfluorescein diacetate succinimidyl ester (CFSE) activation. In their inactivated state, the proliferation rate of the T cells was about 1%. After coactivation with anti-CD3 and anti-CD28 antibodies, the proliferation rate surged to 20%. However, this increase was reversed to 1% when CRAC channel inhibitor BTP2 was applied, indicating that proliferation is controlled by CRAC channel activation. When the T cells were exposed to AC_ext_ following anti-CD3 and anti-CD28 stimulation, their proliferation rate was inhibited in a dose-dependent manner (Figure 2c). This suggests that AC_ext_ can effectively regulate T cell proliferation by modulating CRAC channel activity.

### 2.3. Therapeutic Efficacy of AC_ext_ in an Oxazolone-Induced AD Mouse Model

**Figure 3:**
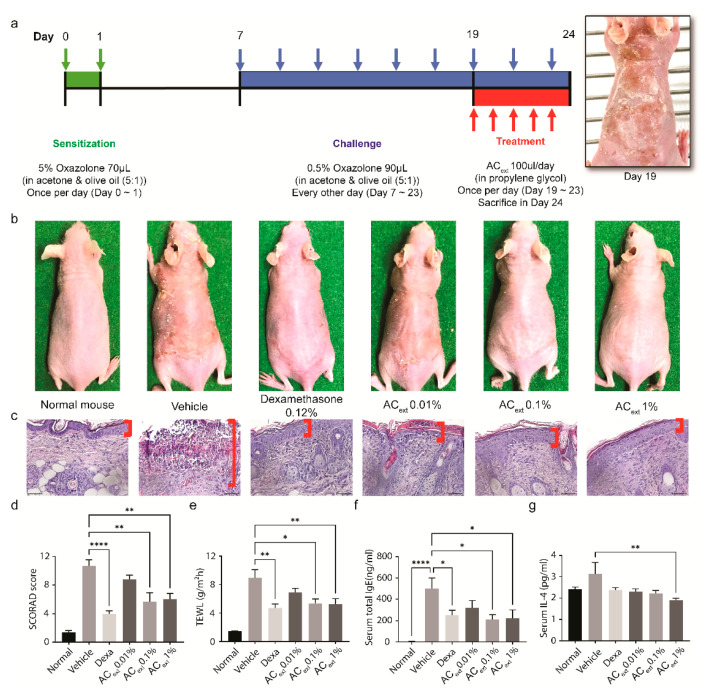
In vivo therapeutic effect of AC_ext_ in AD model (**a**) Schematic timetable for the induction of AD using oxazolone and subsequent treatment with AC_ext_. (**b**) Gross skin photographs showing representative images of skin lesions in the oxazolone-induced AD mouse model, with and without AC_ext_ treatment, demonstrating the extract’s ability to alleviate the skin symptoms of AD. Dexamethasone was used as a positive control. (**c**) Hematoxylin and eosin (H&E) stained sections of the dermis from the mouse model, showing histological differences between treated and untreated groups. (**d**) SCORAD scores of each treated or non-treated group showing quantitative improvement in skin lesion following AC_ext_ treatment. Statistical significance was determined using ANOVA with post-hoc test (* *p* < 0.05, ** *p* < 0.01, **** *p* < 0.0001). (**e**) Total Evaporative Water Loss (TEWL) in treated and untreated groups, indicating barrier function improvement with AC_ext_. (**f**) Serum total IgE levels reflecting the immunomodulatory effect of the extract. (**g**) Serum IL-4 levels highlighting the anti-inflammatory impact of the extract. (ANOVA with post-hoc test, ** *p* < 0.01, **** *p* < 0.0001).

Based on the previous findings, we hypothesized that AC_ext_ could effectively regulate pathological symptoms in vivo. To replicate the pathologic architecture of AD, we utilized oxazolone-induced mouse models [19]. Sensitization was initiated by applying 70 µL of 5% oxazolone to the skin of BALB/c mice for two consecutive days and followed by a challenge phase where 90 µL of 0.5% oxazolone was administered daily for nine days to induce dermatitis. Starting on day 19, topical administration of the extract was applied to evaluate its therapeutic effects. This treatment was compared to a vehicle-treated group and a dexamethasone-treated group (Figure 3a).

The gross examination of the skin lesions indicates that the AC_ext_ markedly ameliorated the symptoms of AD, as evidenced by less severe lesions compared to untreated controls (Figure 3b). Histological analysis using H&E staining shows significant improvements in skin architecture in the AC_ext_ treated group, with reduced epidermal thickening and inflammatory infiltration (Figure 3c). To mimic clinical evaluation in human AD, we measured SCORAD (Scoring Atopic Dermatitis) index in the mouse model [20,21]. Quantitative analysis revealed a significant improvement in mice treated with AC_ext_, demonstrating a notable reduction in dermatitis severity (ANOVA with post-hoc test, ** *p* < 0.01, **** *p* < 0.0001) (Figure 3d). Additionally, the treatment reduced total evaporative water loss (TEWL), indicating an enhancement in the skin barrier function (Figure 3e) [22]. Immunologically, there was a significant decrease in serum total IgE (Figure 3f) and IL-4 levels (Figure 3g) in the AC_ext_ treated group, suggesting a reduction in the allergic and inflammatory responses associated with AD.

### 2.4. Accelerated Skin Barrier Recovery by Topical Application of AC_ext_ in a Mouse Model

The skin serves as a protective barrier against external pathogens and threats by employing a multi-layered defense system. This system encompasses the vigilant action of immune cells, maintenance of chemical balance such as pH and temperature, and the preservation of an intact physical structure to prevent the infiltration of allergens and pathogens into internal tissues and organs [23,24]. In AD patients, symptoms including skin flare, dry skin, itching, and scaly lesions are common, which easily makes the skin barrier become compromised, leading to increased permeability and susceptibility to allergens and pathogens [25].

**Figure 4:**
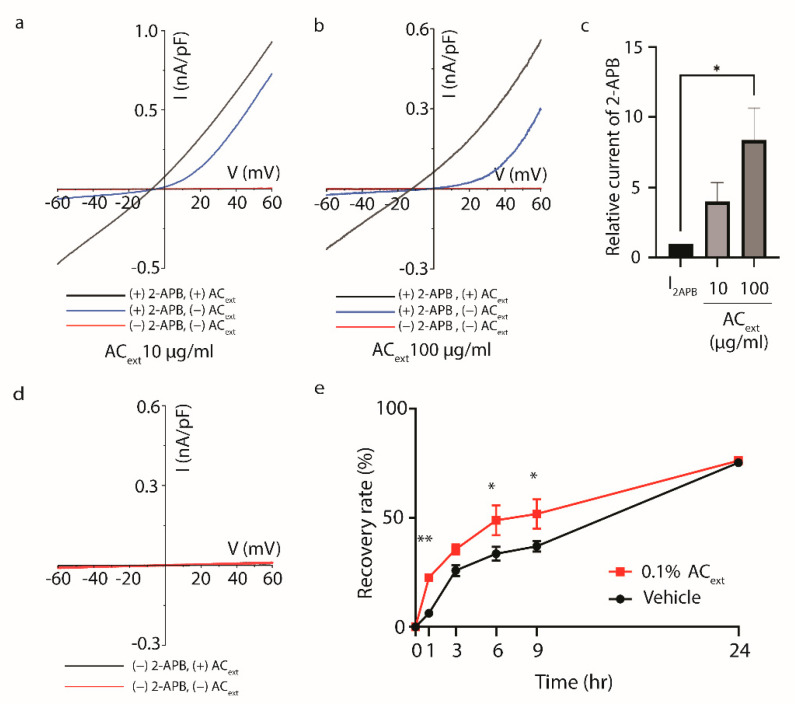
Effects of AC_ext_ on TRPV3 Activity and Oxazolone-Induced Atopic Dermatitis (**a**) TRPV3 current activation by 10 μg/mL AC_ext_ compared with the 100 μg/mL 2-APB activated TRPV3 current. (**b**) TRPV3 current activation by 100 μg/mL M AC_ext_ compared with the 2-APB activated TRPV3 current. (**c**) Comparison of relative current at −60 mV between the control (=1) and the 10 μM and 100 μM treatment groups (mean ± SEM, Student’s *t*-test, * *p* < 0.05). (**d**) Current without TRPV3 activation (2-APB treatment) was not altered by 100 μM AC_ext_ application (**e**) In vivo recovery effect on water loss in damaged skin tissue using AC_ext_ (0.1%) in an AD model (mean ± SEM, two-way ANOVA, post hoc Dunnett’s multiple comparison test vs. vehicle, * *p* < 0.05, ** *p* < 0.01).

Enhancing the proliferation and differentiation of keratinocyte could promote skin barrier recovery. Based on numerous previous studies, it has been reported that TRPV3 (Transient Receptor Potential Vanilloid 3) channel activation induces calcium influx in keratinocytes, thereby promoting their proliferation and differentiation [26,27]. This suggests that TRPV3 could be a potential target for regulating skin regeneration. Therefore, we analyzed whether AC_ext_ modulates the activity of TRPV3. Our results show that AC_ext_ further enhances the activation of TRPV3 induced by 2-APB. However, when treated with AC_ext_ alone, no activation of TRPV3 was observed (Figure 4a–d).

Furthermore, AC_ext_ was shown to accelerate the healing of skin barrier in the in vivo mouse model. Balb/c mice, whose back hair was removed 96 h prior, underwent tape-stripping to remove stratum corneum and create a damaged skin barrier model. Immediately after the removal of the stratum corneum, trans-epidermal water loss (TEWL) was measured. Then, 200 μL of 0.1% AC_ext_ or the control vehicle was applied to two separate groups. TEWL was measured again at 1, 3, 6, and 9 h after application, with reapplication of the substance at each time point. The experimental results show that the recovery rate of the skin barrier significantly accelerated 1 h after topical application of 0.1% AC_ext_ compared to the control group. Additionally, significant skin barrier recovery rates were observed at 6 and 9 h after damage (Figure 4e). This shows the complementary effect of 0.1% AC_ext_ in skin barrier regeneration.

### 2.5. Analysis of HPLC and NMR Fingerprints of AC_ext_

To reveal what chemical substance is related to those pharmacological effects in *Agrimonia coreana*, we performed high-performance liquid chromatography (HPLC) to detect fingerprints of active ingredients from the AC_ext_ used here. To achieve this, the AC_ext_ sample was analyzed at 340 nm under UV absorption conditions, and the two peaks with the largest areas (APH13211, APH13212) were separated (Supplementary Figure S1). Each of the isolated components was measured using ESI-mass spectrometry. For APH13211, in the positive mode, [M+H]+ was observed at m/z 462.9 and [2M+H]+ at *m*/*z* 924.6; in the negative mode, [M−H]- was observed at *m*/*z* 460.9 and [2M−H]- at *m*/*z* 922.6, confirming the molecular weight to be 462 (Supplementary Figure S2). For APH13212, in the positive mode, [M+H]+ was observed at *m*/*z* 446.9 and [2M+H]+ at *m*/*z* 892.6; in the negative mode, [M−H]- was observed at *m*/*z* 444.9 and [2M−H]- at *m*/*z* 890.6, confirming the molecular weight to be 446 (Supplementary Figure S3). Each component was structurally analyzed through NMR, identifying APH13211 as Luteolin-7-O-glucuronide (L7OG) and APH13212 as Apigenin-7-O-glucuronide (A7OG) (Supplementary Figures S4 and S5).

**Figure 5:**
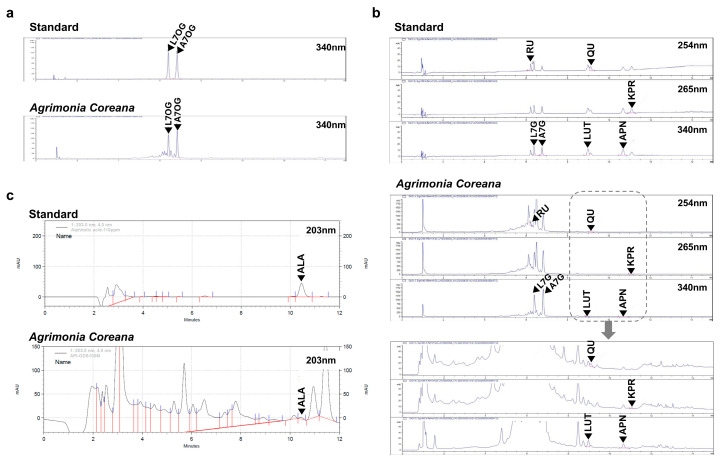
HPLC analysis of *Agrimonia coreana* extract showing marker components (**a**) A7OG (Apigenin-7-glucoside) and L7OG (Luteolin-7-glucoside) are two marker components in the AC_ext_. Inclusion of A7OG is 13.546 ± 0.0045 μg/mg, and that of L7OG is 10.704 ± 0.0212 μg/mg. (**b**) Minor components of the extract are also detected. (RU: rutin, QU:quercetin, KPR:kaempferol, APN:apigenin, LUT:luteolin) (**c**) Compared with standard, alphitolic acid is included in AC_ext_.

The marker components in the extract were quantified using standards, confirming the inclusion of A7OG at 13.546 ± 0.0045 μg/mg and L7OG at 10.704 ± 0.0212 μg/mg (Figure 5a). Additionally, minor components such as rutin, quercetin, kaempferol, apigenin, and luteolin were detected at 254 nm and 265 nm (Figure 5b). However, Apigenin-7-glucoside and Luteolin-7-glucoside were not observed in the samples (Supplementary Figure S6). Furthermore, alphitolic acid, previously reported in our studies, was also confirmed to be present in trace amounts in the sample (Figure 5c) [5].

## 3. Discussion

Our study reveals that *Agrimonia coreana* extract (AC_ext_) effectively modulates CRAC channel activity and T cell responses, offering a promising therapeutic avenue for AD and possibly other inflammatory conditions.

The CRAC channel plays a critical role in calcium signaling associated with T lymphocyte proliferation and is responsible for immune-related pathologies such as allergies, cancers, and inflammatory diseases [14,28,29]. In AD, T cell activation significantly contributes to skin pathologies and is typically managed and treated with immunosuppressive drugs like corticosteroids. However, targeting the CRAC channel by effectively controlling cytokines and cell proliferation via inhibiting calcium signaling pathway presents as a novel alternative [10,12,13].

Agrimony is a widely used herb in traditional medicine, used for various diseases including inflammatory conditions. *Agrimonia coreana* Nakai, a species native to Korea, is particularly noted for its CRAC channel inhibition and also for its anti-inflammatory effect [5]. We further demonstrated that the use of AC_ext_ could be valuable treatment for inflammatory conditions, including AD.

By preventing the calcium influx necessary for T cell activation and proliferation, AC_ext_ effectively reduces the secretion of pro-inflammatory cytokines, such as IL-2, thereby curbing the immune response that leads to AD symptoms. Moreover, the dose-dependent inhibition of T cell proliferation by AC_ext_, as evidenced by flow cytometry and IL-2 assays, reinforces the potential of AC_ext_ as a therapeutic agent targeting immune responses at a cellular level.

AD is also characterized by compromised skin barrier function, facilitating allergen and pathogen penetration, exacerbating inflammation and clinical symptoms. In Figure 4, we observed that while AC_ext_ did not directly activate TRPV3, it enhanced the currents of TRPV3 activated by 2-APB. TRPV3 plays a role in skin barrier formation, with intracellular calcium signaling from its activation promoting keratinocyte proliferation and differentiation via the CaMKII-mediated TGFα/EGFR pathway [26,30]. These findings suggest that AC_ext_ may facilitate the faster recovery of the skin barrier by enhancing TRPV3 activity. However, there are reports indicating that TRPV3 activation may also inhibit keratinocyte proliferation or induce inflammation and pruritus [27,31], necessitating further mechanistic studies on the extract’s role in skin barrier recovery [31].

Our in vivo experiments confirm that topical application of AC_ext_ significantly enhances skin barrier recovery. Reduction in trans-epidermal water loss (TEWL) and improvements in histological markers such as epidermal thickening and inflammatory infiltration provide robust evidence of the beneficial effects of AC_ext_ on skin integrity in atopic dermatitis [32]. The ability of AC_ext_ to accelerate skin barrier recovery suggests its potential as a complementary therapy that can be used alongside conventional treatments to provide more comprehensive management of AD.

In our study, AC_ext_ demonstrated efficacy comparable to dexamethasone, a standard corticosteroid treatment, in reducing AD symptoms in a mouse model. This finding is significant given the known possible adverse effects associated with long-term corticosteroid use, such as skin atrophy [33] and systemic side effects [34]. The lack of such adverse effects with AC_ext_ positions it as a safer alternative for chronic use in AD management. However, detailed safety evaluations in human subjects are required to fully establish the risk–benefit profile. For example, as *Agrimonia coreana* is a member of the Rosaceae family, the risk of the plant-derived product acting as a cross-reactive allergen should be further elucidated, especially in people with Rosaceae species food allergy [35,36].

Further detailed studies are also needed to elucidate the precise molecular pathways affecting CRAC channels and skin barrier function. This could involve proteomic and genomic approaches to identify specific targets and pathways modulated by AC_ext_ as many pathways could be related in barrier formation or defect [1,25,37]. Additionally, it is essential to identify the bioactive metabolites within AC_ext_ and their synergistic effects with other molecules. Also, determining the effective concentrations of the metabolites could provide deeper insights into the mechanism of pharmacologic action. Given its anti-inflammatory and barrier-enhancing properties of AC_ext_, exploring AC_ext_’s effects on other inflammatory and allergic conditions could broaden its therapeutic applications. Also, it would be invaluable to compare AC_ext_ with other AD treatments that share similar pathway regulation mechanisms, such as topical calcineurin inhibitors (TCIs). TCIs are known for their comparable efficacy to standard corticosteroid treatments and are widely used by AD patients worldwide [38]. Positive results from such comparisons would support AC_ext_ as a viable therapeutic option and provide an alternative treatment choice with clear clinical benefits for many patients.

In conclusion, *Agrimonia coreana* shows promising potential as a novel therapeutic agent for AD, offering a dual approach by modulating immune responses and enhancing the skin barrier function. Continued research and clinical validation of its safety in humans are necessary to fully harness its therapeutic benefits and integrate it into standard treatment protocols for inflammatory skin diseases.

## 4. Materials and Methods

### 4.1. Cell Culture

Human Embryonic Kidney 293 T (HEK293T) cell and Jurkat T cells were purchased from the American Type Culture Collection (ATCC, Manassas, VA, USA). HEK293T cells were maintained in the incubator setting at 37 °C and 10% CO_2_. The culture medium consisted of Dulbecco’s modified Eagle’s medium (DMEM, Welgene, Gyeongsan, Republic of Korea), 10% Fetal Bovine serum (FBS, Welgene) and 1% Penicillin/Streptomycin (P/S; GE Healthcare, Chicago, IL, USA). Jurkat T cells were cultured in the incubator setting at 37 °C with 5% CO_2_, and the culture medium was RPMI1640 (Gibco, Grand Island, NY, USA) with 10% FBS and 1% P/S.

### 4.2. Transient Transfection

To measure the CRAC current, HEK293T cells were co-transfected with the human ORAI1 (hORAI1) and human STIM1 (hSTIM1) vector. Transfection was performed using Turbofect (Thermo Scientific, Waltham, MA, USA) according to the manufacturer’s protocol. Green fluorescence protein (pEGFP-N1, Life Technologies, Carlsbad, CA, USA) was co-transfected at a 10:1 ratio to mark the transfected cells. Previous study has shown the detailed method of transient transfection [39].

### 4.3. Electrophysiology

CRAC current was measured with the transient transfected HEK293T cells. The current was recorded using Axopatch 200B (Molecular Devices, Sunnyvale, CA, USA) and Digidata 1440A (Molecular Devices). We analyzed the raw data with pCLAMP 10.4 (Molecular Devices), Origin 8 (Microcal, Northampth, MA, USA), and GraphPad prism 10 (GraphPad, La Jolla, CA, USA). A previous paper has reported the recording and analysis of the whole-cell patch clamp for I_CRAC_ (I_ORAI1_) [40].

### 4.4. High Performance Liquid Chromatography (HPLC)

The components of *Agrimonia coreana* extract (AC_ext_) were analyzed using HPLC (1290 Series; Agilent Technologies, Santa Clara, CA, USA) at the Korea Basic Science Institute (Seoul, Republic of Korea). AC_ext_ (10 mg/mL; 10 μL) was injected into a YMC-Triart C18 column (100 mm × 2.1 mm, 5 μm; YMC America, Devens, MA, USA), separated, and detected for its chemical constituents at the wavelengths of 254 nm, 265 nm, and 340 nm. The column temperature was maintained at 25 °C. The mobile phases were 0.05% formic acid (A) and 0.05% formic acid/acetonitrile (B) used at a flow rate of 0.3 mL/min. The gradient conditions were as follows: 5% B at 0 min; 5–10% B for 3 min; 10–30% B for 2 min; 30–40% B for 5 min; 40–90% B for 2.5 min; and equilibration with 90% B for 2.5 min.

### 4.5. Preparation of 70% Ethanolic Extract of Agrimonia coreana

Dried *Agrimonia coreana* was pulverized to prepare 30 kg of material. The extraction solvent used was 70% ethanol, added to 20 times the weight of the primary material (600 L). The mixture was subjected to circulation extraction at approximately 70 °C for 4 h. The primary extract was filtered using filter paper (Advantec, 150 mm, NO. 5B) and subsequently cooled. The filtered extract was concentrated to approximately 29–33 Brix at 55 °C to 60 °C. The concentrate was then mixed with 95% ethanol in a 1:1 ratio and introduced into a column containing HP20 resin for adsorption. The adsorbed extract was sequentially eluted with 50% ethanol and 95% ethanol. The eluate was concentrated to approximately 10 Brix at temperatures below 60 °C. The concentrated extract was sterilized and then freeze-dried.

### 4.6. Interleukin-2 (IL-2) Cytokine Assay

To induce the secretion of Interleukin-2 (IL-2), Jurkat T cells were co-stimulated with anti-CD3 (Peprotech, Rocky Hill, NJ, USA) and anti-CD28 antibody (Peprotech). First, 5 μg/mL anti-CD3 was added to a 96-well plate for 50 μL/well to coat the plate for 3 h at 37 °C, followed by DPBS washing for three times. Next, Jurkat T cells were seeded at a density of 5 × 10^5^ cells/well. Additionally, 2 μg/mL anti-CD28 per well was coated and incubated for 72 h similarly in the incubator at 37 °C with 5% CO_2_. Finally, the supernatant of the culture media was collected and diluted 1:2. The total amount of IL-2 secreted by Jurkat T cells was measured using the IL-2 ELISA kit (Peprotech), according to the manufacturer’s protocol.

### 4.7. Human Primary CD4+ T Lymphocyte Isolation

All procedures involving human blood were approved by the Institutional Review Board (IRB) of Dongguk University College of Medicine (2017-07-003 IRB). Peripheral blood samples were obtained from healthy voluntary donors. Peripheral blood mononuclear cells (PBMCs) were isolated using a Ficoll-Paque Plus medium (GE Healthcare, Chicago, IL, USA) through density gradient centrifugation. Naïve human T lymphocytes were then isolated using a CD4+ T cell isolation kit (Miltenyi Biotec, Bergisch Gladbach, Germany) according to the manufacturer’s protocol.

### 4.8. T Cell Proliferation Assay

Cellular proliferation was assessed by labeling purified CD4+ T cells with carboxyfluorescein succinimidyl ester (CFSE), and they were analyzed by fluorescence-activated cell sorting. The specific methods are described in a previous study [40].

### 4.9. Trans-Epidermal Water Loss (TEWL) Assay

All animal experiments in this study were approved by the Institutional Animal Care and Use Committee (IACUC) of Dongguk University Ilsan Hospital (2016-02145). The handling of mice adhered to the guidelines for animal experiments and related activities at Dongguk University. Female BALB/c mice aged 7 weeks were from Orientbio, Kyunggido, Republic of Korea.

To conduct a skin barrier recovery test after tape stripping, the dorsal hair of the mice was removed using hair removal cream (Veet™, Reckitt Benckiser, Slough, UK). The tape-stripping method followed a previously documented procedure. Different concentrations of APH2O (1, 10, and 30 mg/mL) were prepared by dissolving the extract in normal saline from Daehan Pharma in Seoul, Republic of Korea. The measurement of the skin barrier function, barrier disruption, and subsequent treatment with APH2O or the vehicle (normal saline) were performed under anesthesia using 3% isoflurane (Ifran™, Hana Pharma, Seoul, Republic of Korea).

Due to variations in stratum corneum thickness among mice, the dorsal skin on both flanks was repeatedly stripped using cotton plaster until trans-epidermal water loss (TEWL) reached approximately 140 g/(m^2^·h). The average TEWL after tape stripping was 140 ± 15 g/(m^2^·h) (n = 24). Following the tape stripping, 100 μL of APH2O in saline (1, 10, and 30 mg/mL) or normal saline was applied to the dorsal skin of each mouse (approximately 5 cm^2^). Thus, the final applied concentrations of APH2O were 0.02, 0.2, and 0.6 mg/cm^2^. The treated dorsal skin was then covered with plastic wrap for 15 min to aid absorption. TEWL was subsequently measured at the same site at 0, 1, 3, 6, 10, 24, and 30 h after barrier disruption using a vapometer (SWL5051, Delfin Technologies Ltd., Kuopio, Finland). The ambient temperature was maintained at 20 ± 1 °C, with humidity at 50 ± 5%. The percentage barrier recovery was calculated using the formula: % barrier recovery = [(TEWL at 0 h − average TEWL of APH2O-treated group at the indicated time point)/(TEWL at 0 h − average TEWL of the normal group at the indicated time point)] × 100. Data are presented as mean ± standard error of the mean (SEM). Statistical significance between groups was determined using two-way analysis of variance (ANOVA). For significant effects, individual groups were compared using Dunnett’s post-hoc test, with *p*-values < 0.05 considered statistically significant for all comparisons. Two-way ANOVA and post-hoc tests were performed using GraphPad Prism 6.0 software (GraphPad, La Jolla, CA, USA).

### 4.10. Statistics

For general statistical analysis, Origin 8 (Microcal, Northampth, MA, USA), and GraphPad prism 10 (GraphPad, La Jolla, CA, USA) were utilized. Data are presented as mean ± standard error of the mean (SEM). For experiments evaluating the impact of a single extract, Student’s *t*-test was employed to compare two groups. Experiments assessing the effects of multiple extract concentrations, IL-2 analysis, and those verifying T cell proliferation inhibition were analyzed using one-way ANOVA, followed by Bonferroni multiple comparison post-hoc tests. Statistical analysis of β-Hexosaminidase activity involved one-way ANOVA, followed by Newman–Keuls multiple comparison post-hoc tests. Statistical significance was defined as *p* < 0.05.

## 5. Conclusions

Our study demonstrates that AC_ext_ is a promising candidate for an alternative therapeutics for AD. By inhibiting CRAC channel activity, AC_ext_ could reduce T cell proliferation and the secretion of cytokines to regulate the allergic response. Furthermore, AC_ext_ enhances the activation of the TRPV3 channel, promoting keratinocyte proliferation and differentiation, which are crucial for skin barrier repair. In vivo evidence shows that topical AC_ext_ improves skin lesions and reduces inflammation and barrier dysfunction in an oxazolone-induced AD mouse model, with effects comparable to those of corticosteroid dexamethasone through dual ion channel activation mechanism. Our findings are supported by the identification of key active compounds, Luteolin-7-O-glucuronide and Apigenin-7-O-glucuronide, through HPLC and NMR analysis, underscoring the extract’s pharmacological potential.

With AC_ext_ showing a favorable result, further studies including clinical trials with safety profiles are needed to fully establish it as a treatment. In conclusion, AC_ext_ shows the potential for a therapeutic agent for AD, offering both immunomodulatory and skin barrier-enhancing clinical benefits.

## Data Availability

The data that support this study are available from the corresponding author upon reasonable request.

## Supplementary Materials

The following supporting information can be downloaded at: https://www.mdpi.com/article/10.3390/ijms25168894/s1.
