# Supplementary material for: Agrimonia coreana Extract Exerts Its Therapeutic Effect through CRAC Channel Inhibition for Atopic Dermatitis Treatment
Source: Int J Mol Sci. 2024 Aug 15;25(16):8894. doi: 10.3390/ijms25168894 (PMC11355045; doi:10.3390/ijms25168894)
Supplement: Supplementary file 1 [file ijms-25-08894-s001.zip › ijms-3145108-supplementary.pdf]

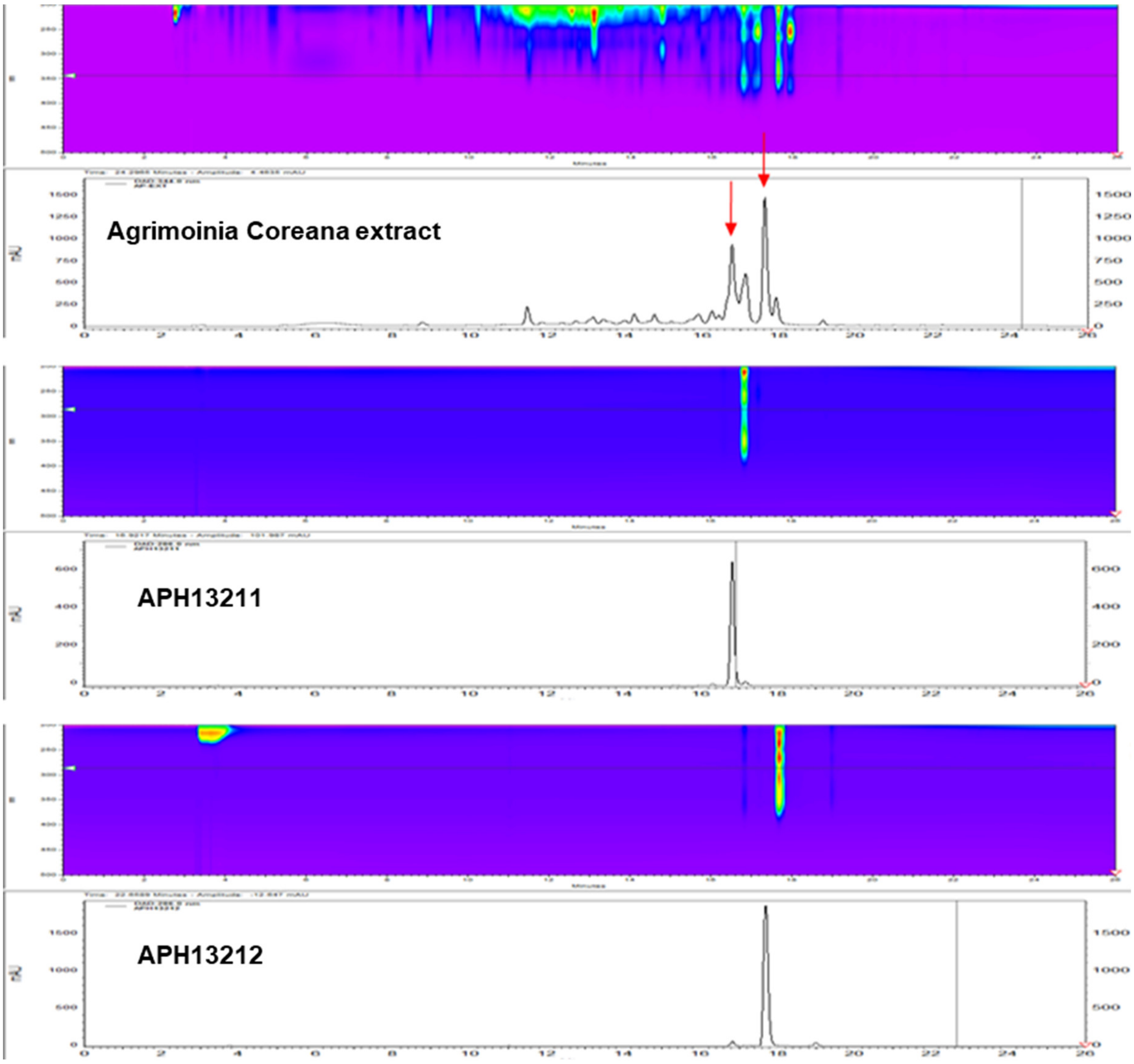

Supplementary Figure S1. HPLC of *A. coreana extract*

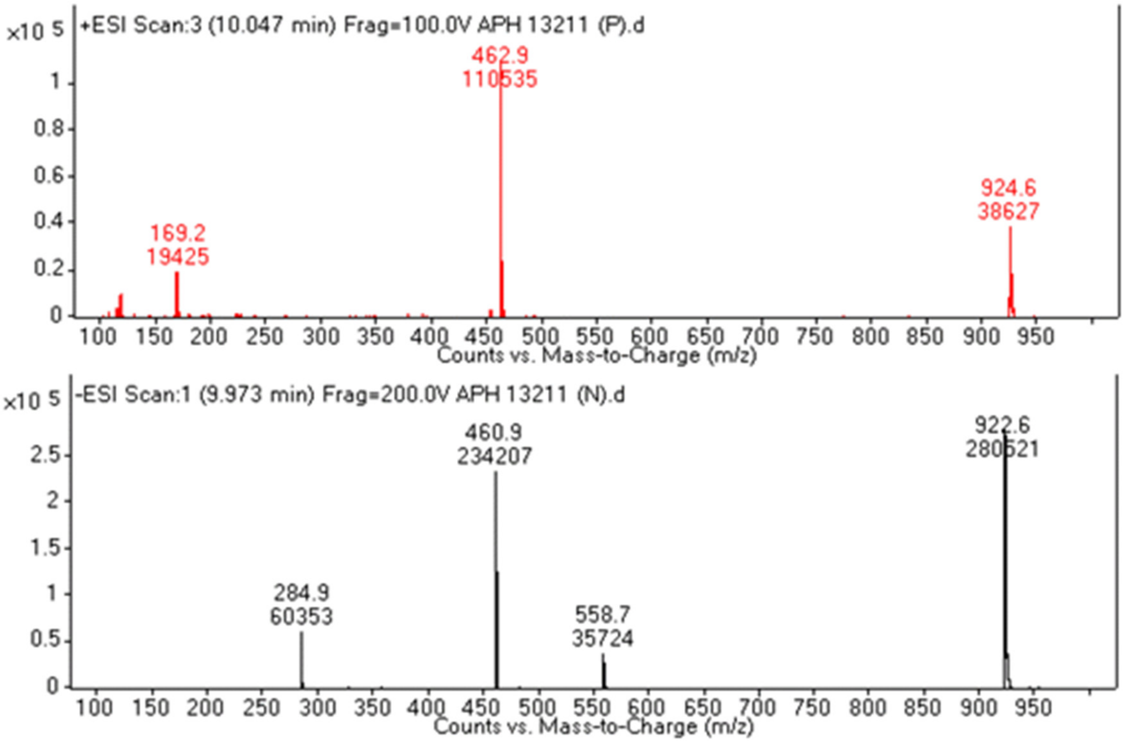

Supplementary Figure S2. ESI-mass spectrum of APH13211. (upper panel; positive mode, bottom; Negative mode)

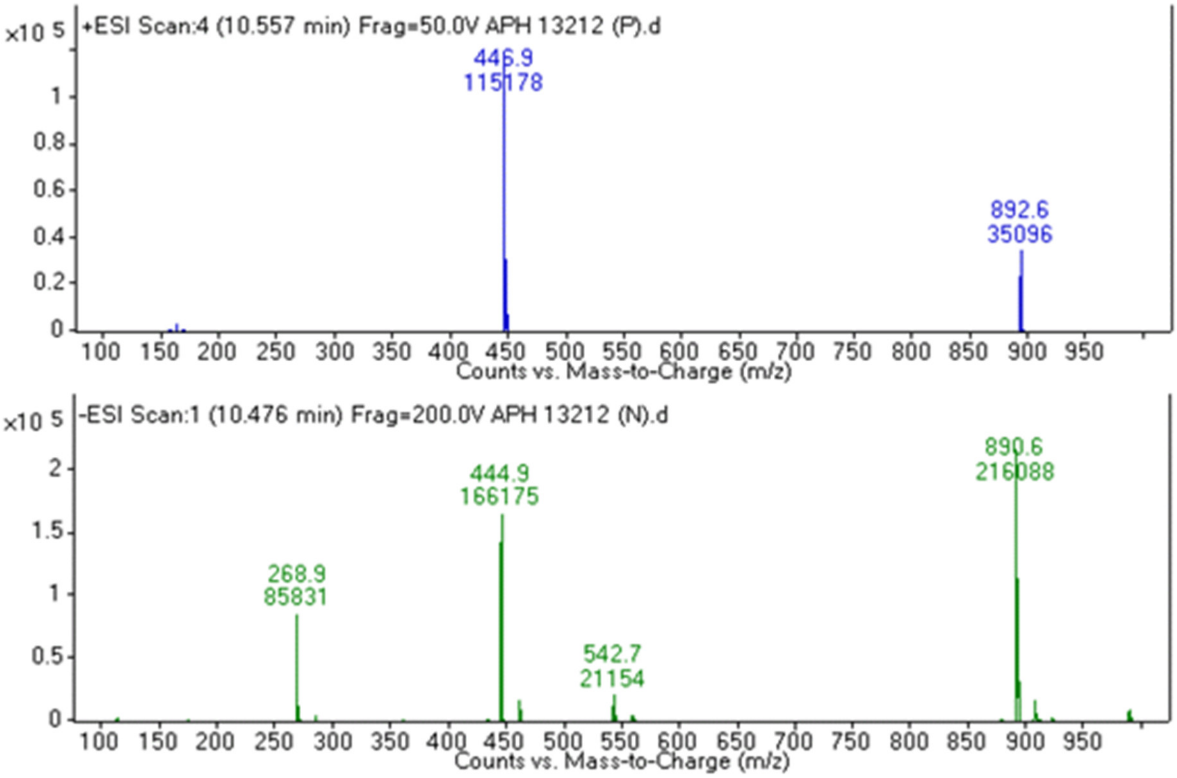

Supplementary Figure S3. ESI-mass spectrum of APH13212. (upper panel; positive mode, bottom; Negative mode)

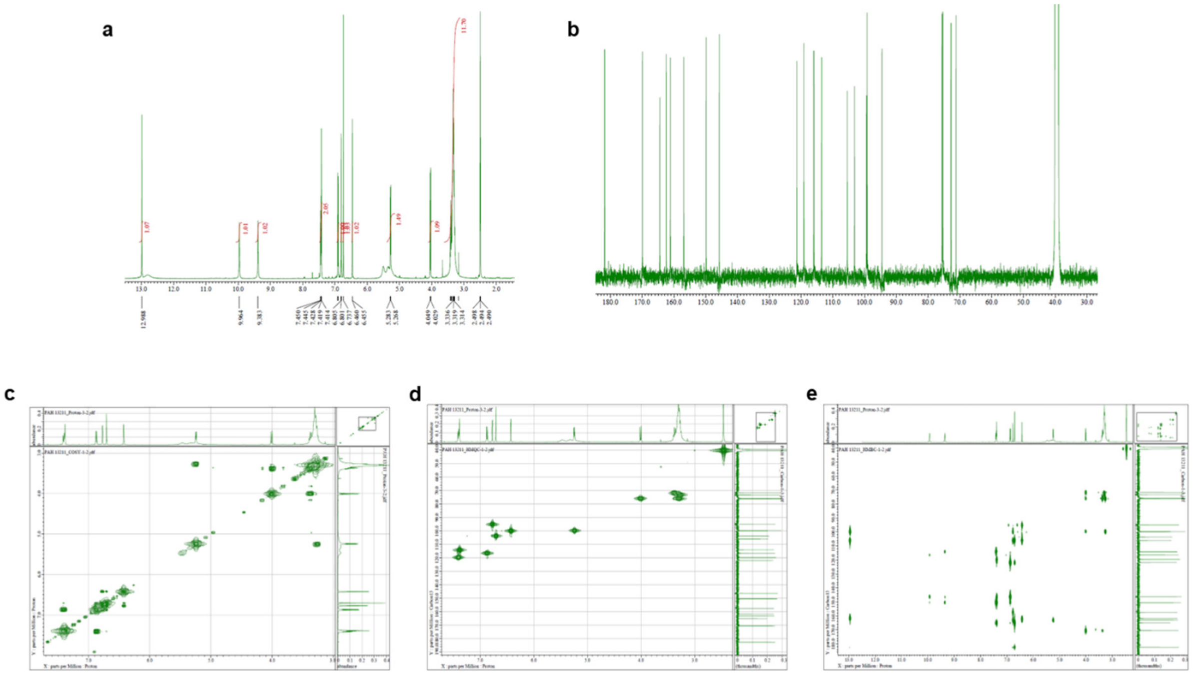

Supplementary Figure S4. NMR of APH13211. (**a**) 1H NMR spectrum, (**b**) 13C NMR spectrum, (**c**) 1H-1H COSY spectrum, (**d**) HMQC spectrum, (**e**) HMBC sepctrum

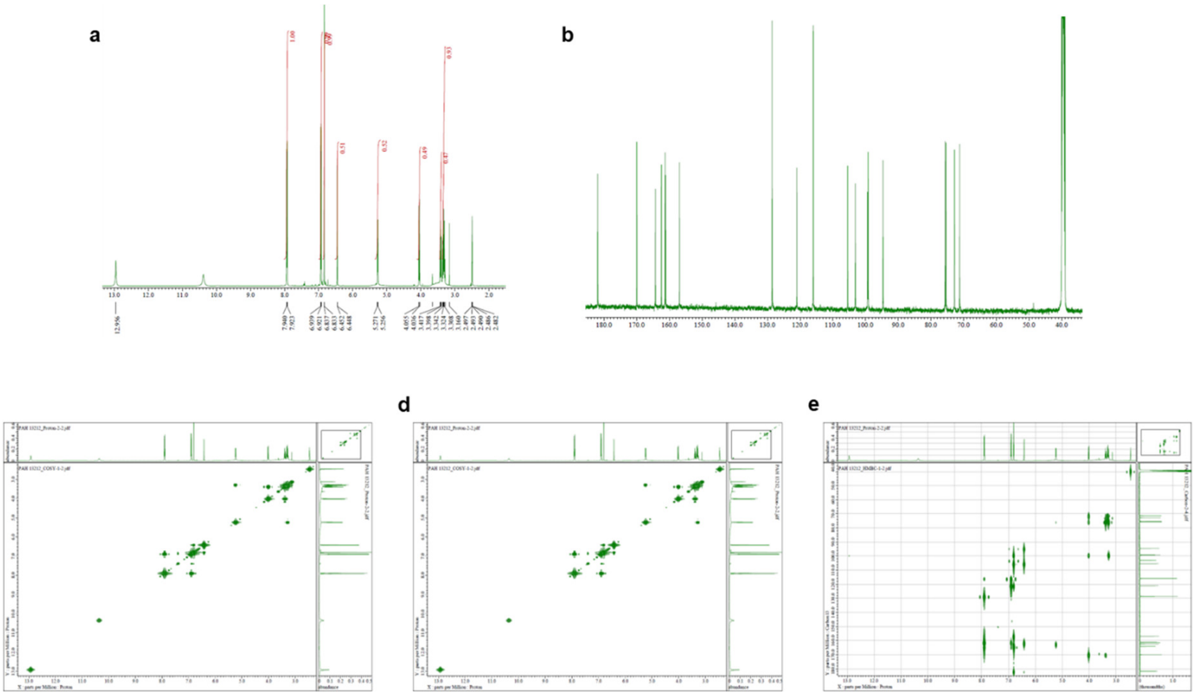

Supplementary Figure S5. NMR of APH13212. (**a**) 1H NMR spectrum, (**b**) 13C NMR spectrum, (**c**) 1H-1H COSY spectrum, (**d**) HMQC spectrum, (**e**) HMBC sepctrum

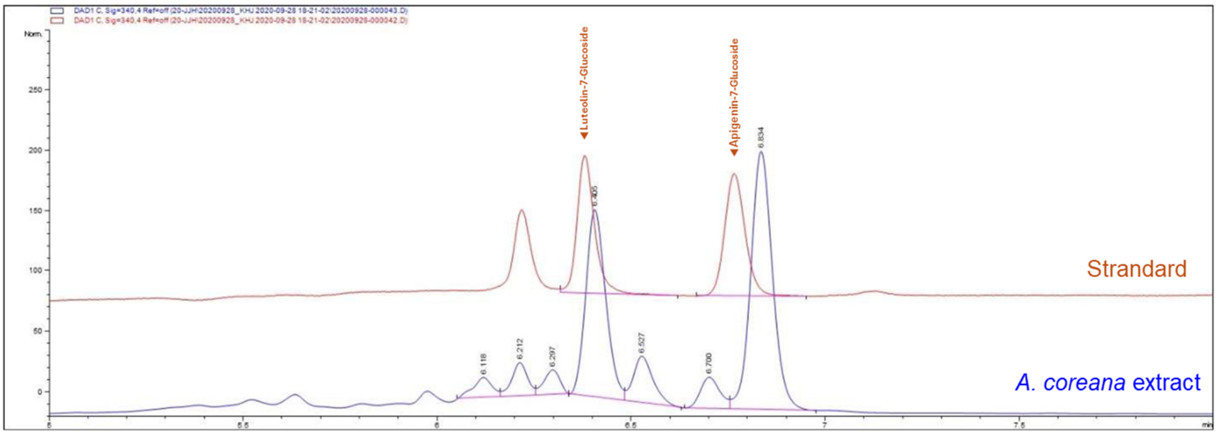

Supplementary Figure S6. HPLC of *A. coreana extract*
